# Supplementary material for: Structure-guided design of endosomolytic chloroquine-like lipid nanoparticles for mRNA delivery and genome editing
Source: Nat Commun. 2025 May 7;16:4241. doi: 10.1038/s41467-025-59501-y (PMC12058976; doi:10.1038/s41467-025-59501-y)
Supplement: Supplementary file 1 — Supplementary Information [file 41467_2025_59501_MOESM1_ESM.pdf]

Structure-guided design of endosomolytic chloroquine-like lipid nanoparticles for mRNA delivery and genome editing

Zhen Liu[1,†], Jiacai Wu[2,†], Ning Wang[2], Yongqi Lin[2], Ruiteng Song[1], Min Zhang[3] and Bin Li[1,2,*]

[1]Department of Infectious Disease, Shenzhen People's Hospital, The Second Clinical Medical College of Jinan University, Shenzhen 518020, China.

[2]School of Medicine, Southern University of Science and Technology, Shenzhen, 518055, China.

[3]Department of Ophthalmology, Shenzhen People's Hospital, The Second Clinical Medical College, Jinan University, Shenzhen 518020, China.

[†]These authors contributed equally: Zhen Liu, Jiacai Wu.

[*]Corresponding author: libin@mail.sustech.edu.cn (B. Li).

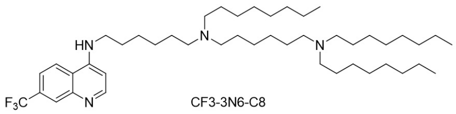
CF3-3N6-C8

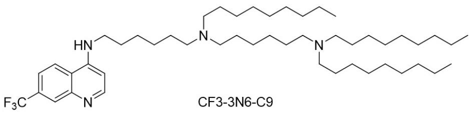
CF3-3N6-C9

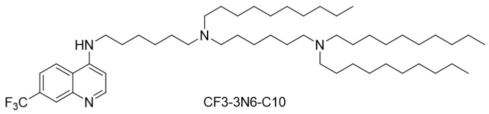
CF3-3N6-C10

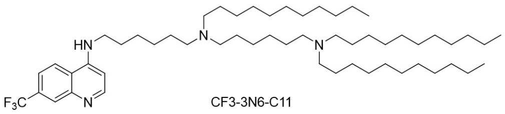
CF3-3N6-C11

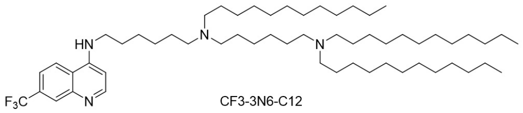
CF3-3N6-C12

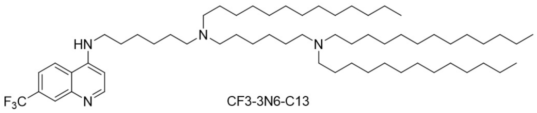
CF3-3N6-C13

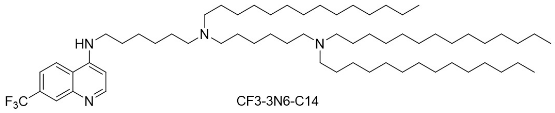
CF3-3N6-C14

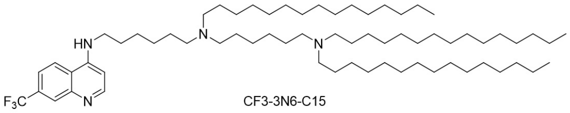
CF3-3N6-C15

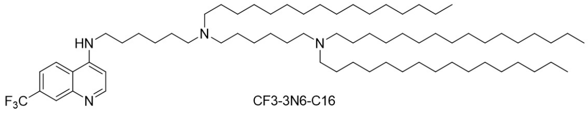
CF3-3N6-C16

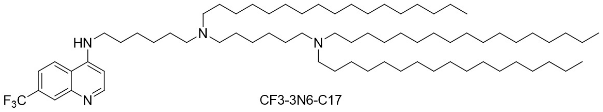
CF3-3N6-C17

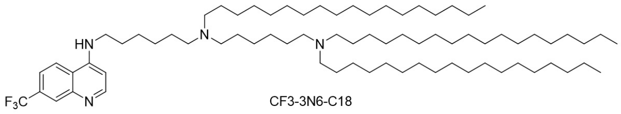
CF3-3N6-C18

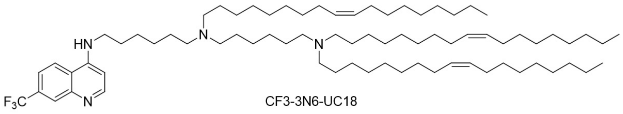
CF3-3N6-UC18

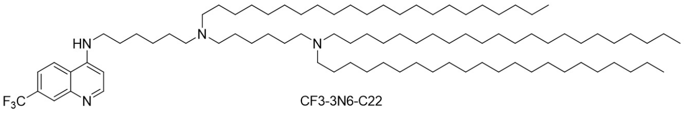
CF3-3N6-C22

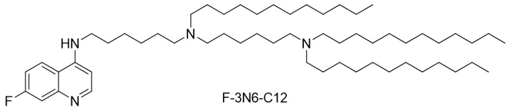
F-3N6-C12

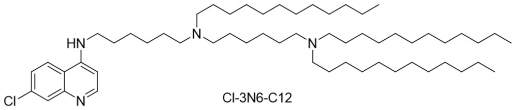
Cl-3N6-C12

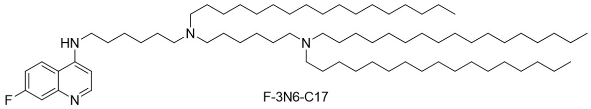
F-3N6-C17

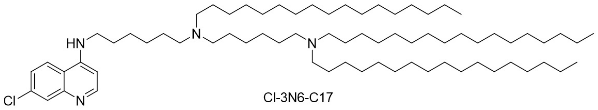
Cl-3N6-C17

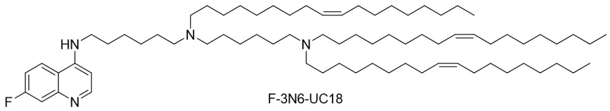
F-3N6-UC18

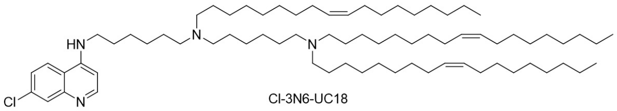
Cl-3N6-UC18

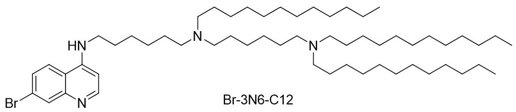
Br-3N6-C12

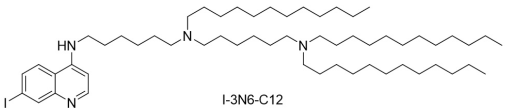
I-3N6-C12

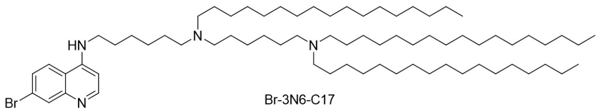
Br-3N6-C17

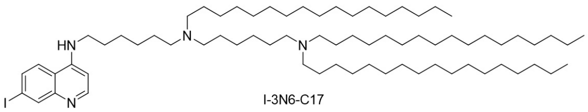
I-3N6-C17

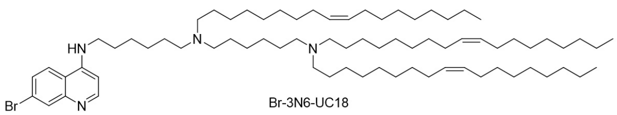
Br-3N6-UC18

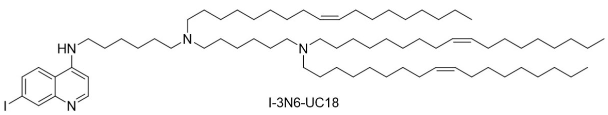
I-3N6-UC18

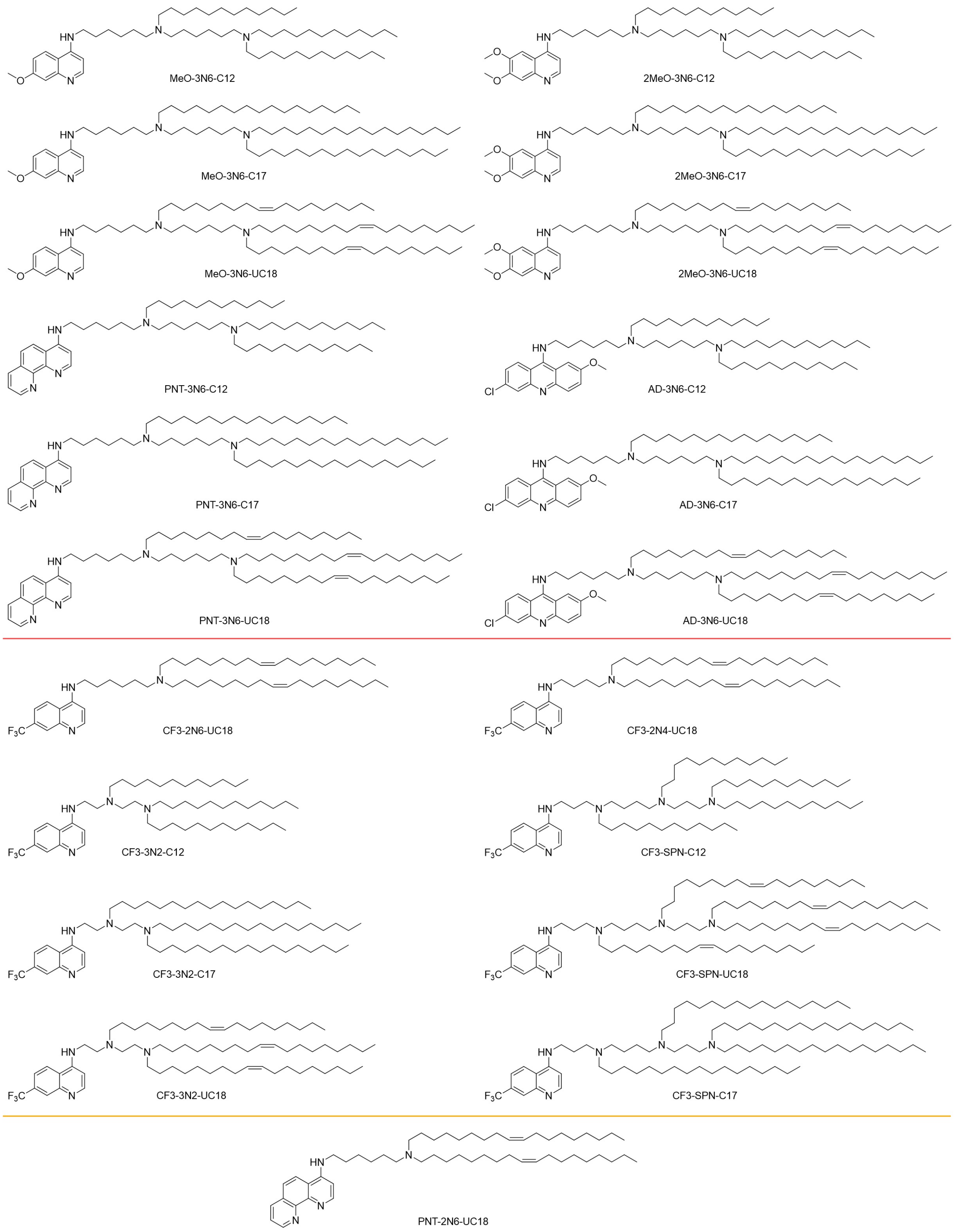

Supplementary Fig. 1 | Chemical structures of chloroquine-like lipids used in this study. PNT-2N6-UC18 was designed as a control lipid in the endosomal escape assay.

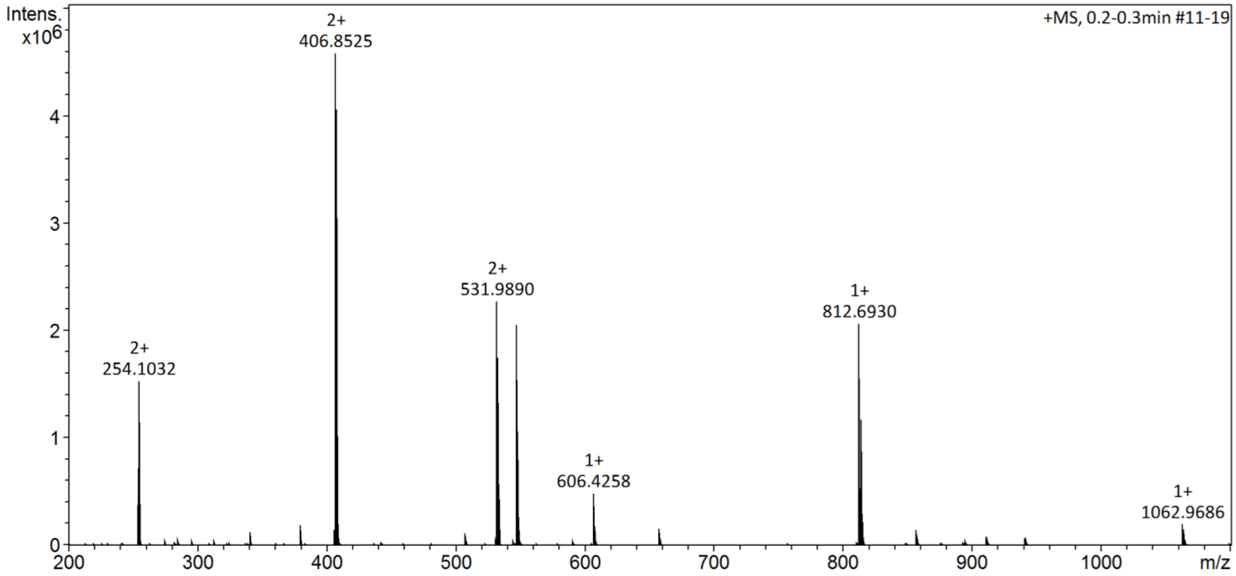

Supplementary Fig. 2 | High resolution mass spectrometry (HR-MS) of CF3-2N6-UC18. CF3-2N6-UC18 was dissolved in methanol prior to measurement.

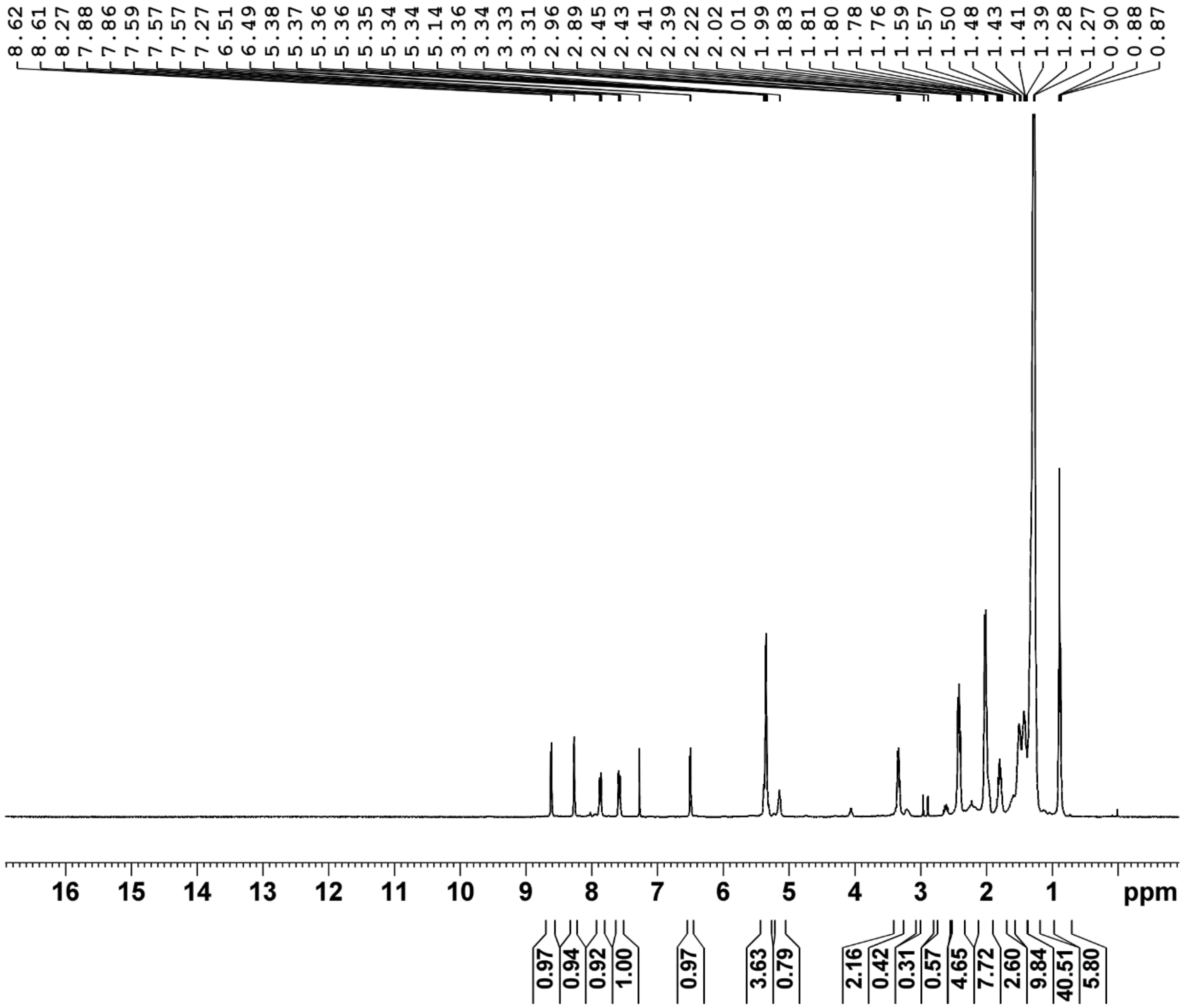

Supplementary Fig. 3 | ^{1}H-NMR spectrum of CF3-2N6-UC18. NMR measurement was carried out in chloroform-d.

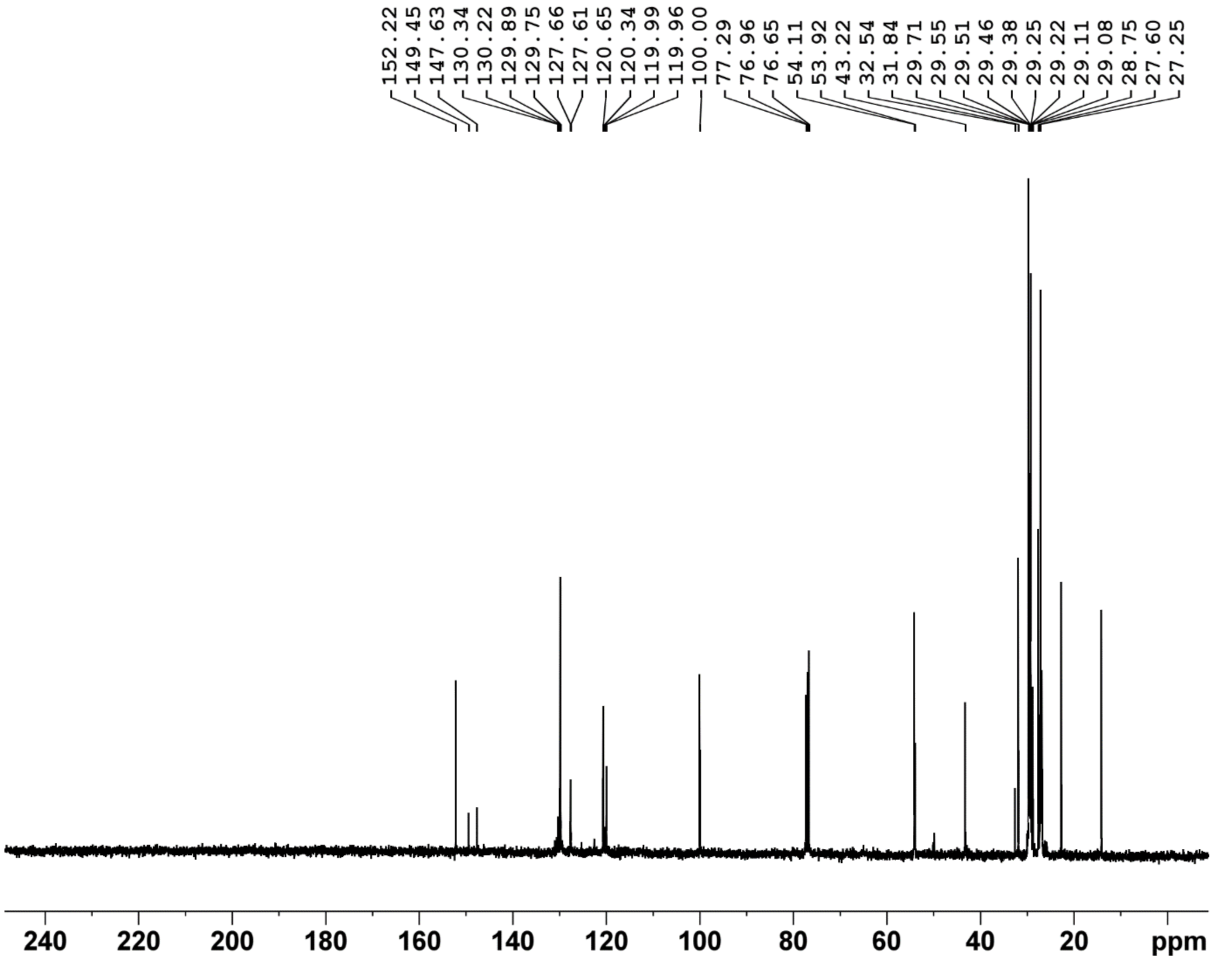

Supplementary Fig. 4 | ^{13}C-NMR spectrum of CF3-2N6-UC18. NMR measurement was carried out in chloroform-d.

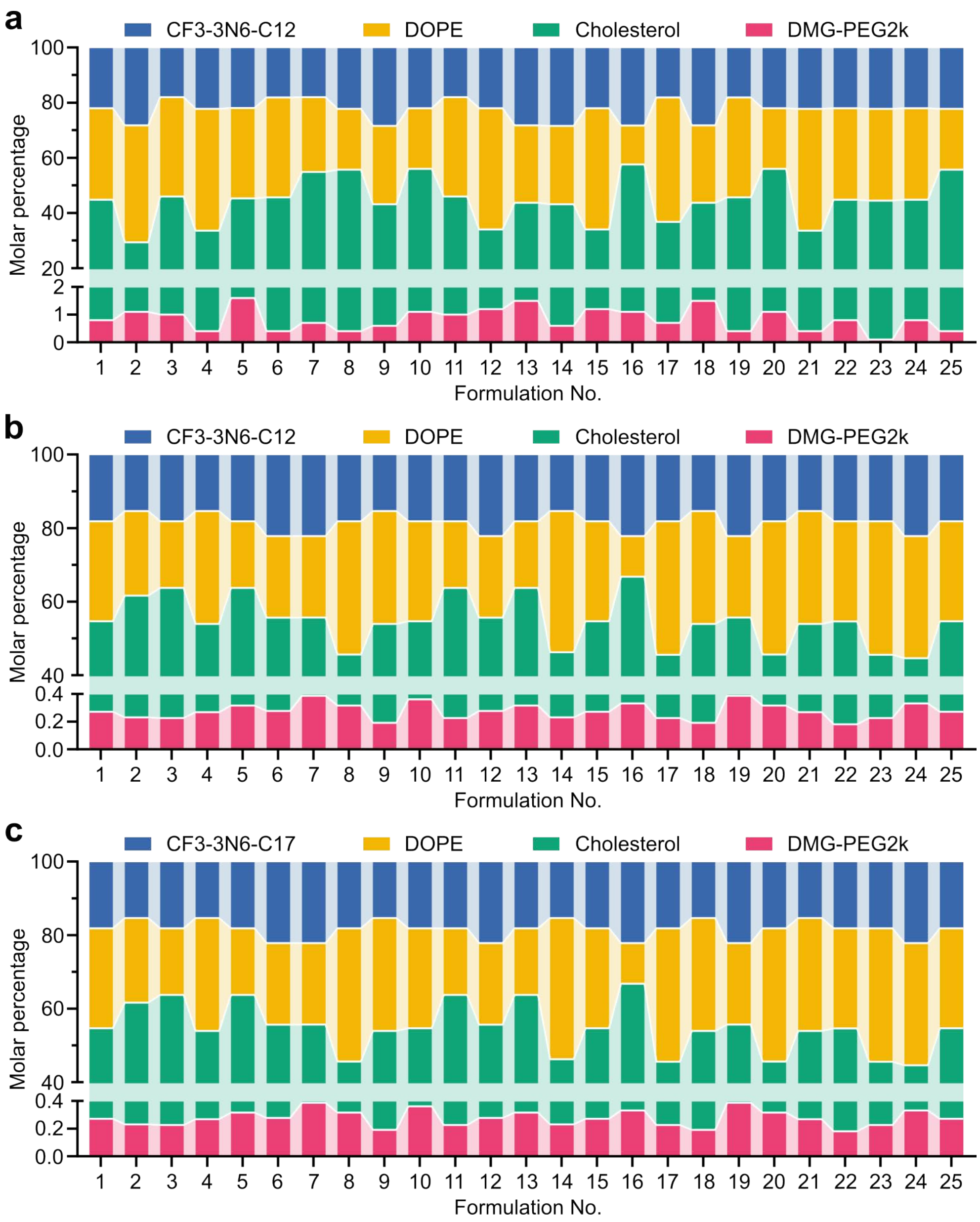

Supplementary Fig. 5 | The molar percentages of four components used in formulation optimization. a-c Twenty-five formulations in the 1st (**a**), 2nd (**b**), and 3rd (**c**) rounds of optimization were generated by combining five levels (molar percentages) of each component with the CCD program.

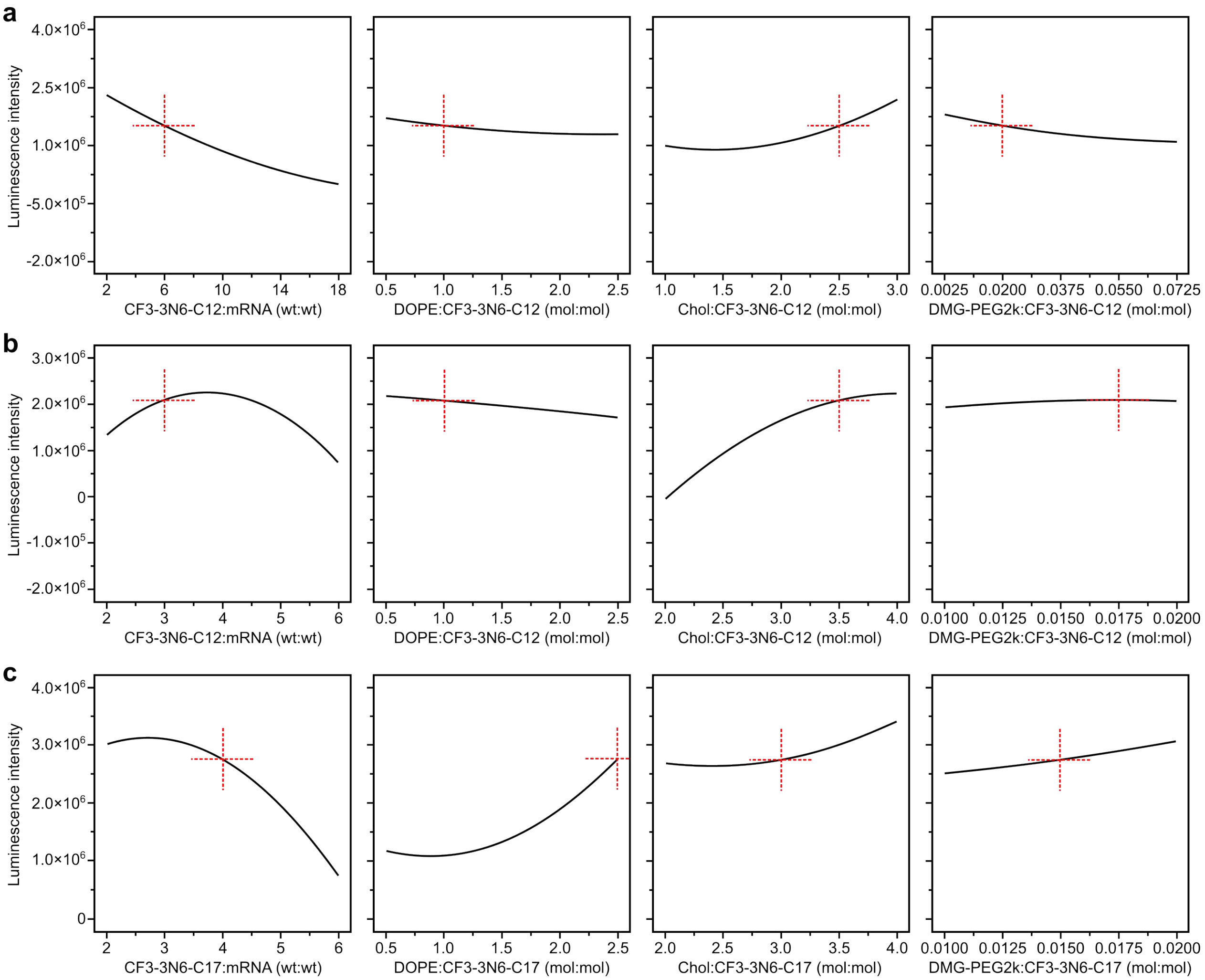

Supplementary Fig. 6 | Effect of composition ratios on mRNA formulations' delivery efficiency. a-c Trends towards delivery efficiency within the experimental range were predicted by the CCD program based on the measured luminescence intensity of each formulation in the 1st (**a**), 2nd (**b**), and 3rd (**c**) rounds of optimization. Red cross indicated the level (ratio) of four factors (Cll:mRNA (wt:wt), DOPE:Cll (mol:mol), Chol:Cll (mol:mol), and DMG-PEG2k:Cll (mol:mol)) of the optimum formulation measured.

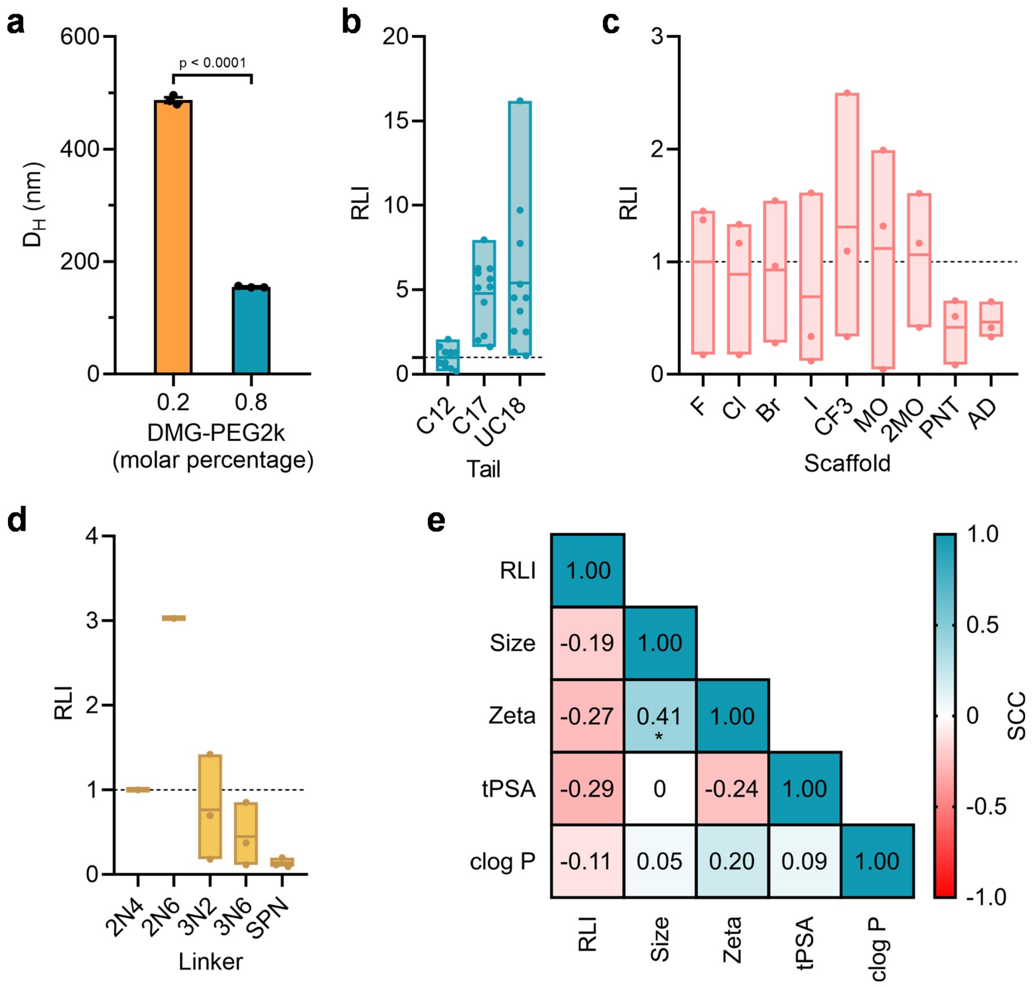

Supplementary Fig. 7 | Effect of formulation parameters on particle characteristics. a Effect of molar percentage of DMG-PEG2k on the hydrodynamic size of CF3-3N6-UC18 LNPs. Data are presented as mean ± SEM (n = 3 biological replicates, unpaired, two-tailed Student's t-test). **b-d** Summary of delivery efficiency of formulations from Fig. 3**e**, classified according to the tails (**b**), scaffolds (**c**), and linkers (**d**) of CIls. Luminescence intensity of each group was normalized to the first item. Floating bars showing the relative luminescence intensity (RLI) of each group (the first group was used for normalization); middle line, mean; box limits, minimum and maximum. **e** Spearman's correlation matrix of relevant variables including RLI, size, zeta, tPSA, and clog P. SCC, Spearman's correlation coefficient. * indicates p = 0.013. Unlabeled data indicates not significant.

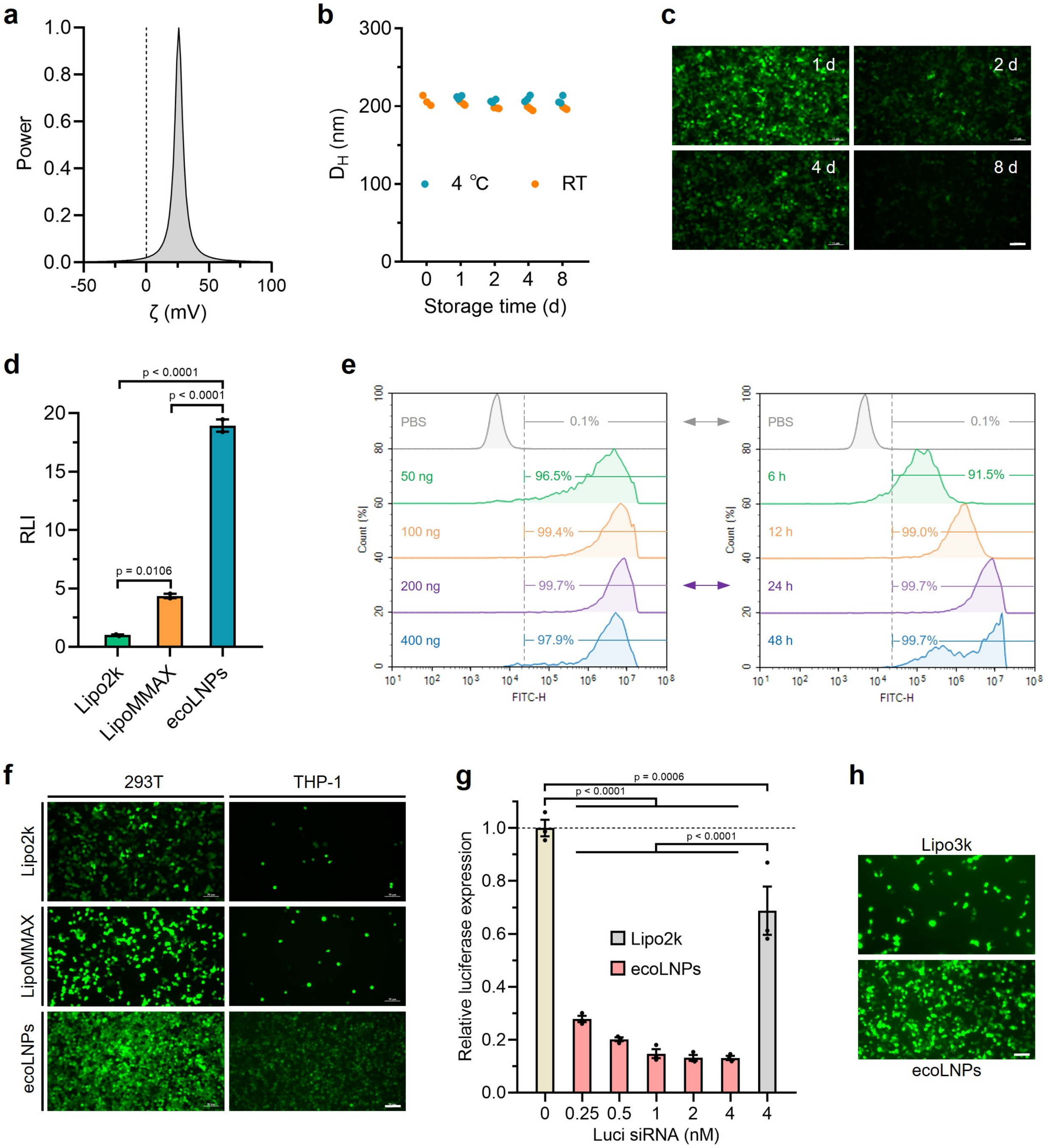

Supplementary Fig. 8 | Zeta potential, stability, and in vitro delivery activity of ecoLNPs. a The zeta potential (ζ) of ecoLNPs determined by dynamic light scattering. **b** The z-average hydrodynamic diameter (D_H) of ecoLNPs during storage at 4 °C or room temperature (RT). (*n* = 3 biological replicates, one-way ANOVA with Tukey's multiple comparison test). **c** In vitro delivery performance of ecoLNPs during storage at RT. ecoLNPs containing 200 ng of eGFP mRNA were

stored at RT for different storage times and their in vitro delivery performance was estimated by the intensity of green fluorescence originating from ecoLNPs-treated 293T cells. Scale bar, 50 μm. **d** Comparison of delivery activity mediated by ecoLNPs and two commercially transfection reagents (Lipo2k and LipoMMAX). Luminescence intensity was normalized to that of Lipo2k (n = 2 biological replicates, one-way ANOVA with Tukey's multiple comparison test). **e** Quantitative analysis of GFP-positive 293T cells after 24 h treatment with exposed to varying doses of eGFP mRNA-loaded ecoLNPs (left panel) or at different time intervals (200 ng, right panel) by flow cytometry. PBS-treated cells were used to differentiate positive and negative events. Both panels shared the PBS-treated group and 200 ng & 24h-treated group. **f** Representative fluorescence microscopy images of 293T and THP-1 cells after 24 h treatment with 200 ng of eGFP mRNA-loaded ecoLNPs. Lipo2k and LipoMMAX were used as the positive groups. Scale bar, 50 μm. **g** Knockdown of luciferase expression via siRNA-loaded ecoLNPs. Lipo2k was used a positive control. Luminescence intensity was normalized to that of PBS treated cells (0 nM) (n = 3 biological replicates, one-way ANOVA with Tukey's multiple comparison test). **h** Representative fluorescence microscopy images of 293T cells after 24 h treatment with eGFP plasmid-loaded ecoLNPs. Lipo3k was used as a positive control. Scale bar, 50 μm. For all relevant panels, data are presented as mean ± SEM.

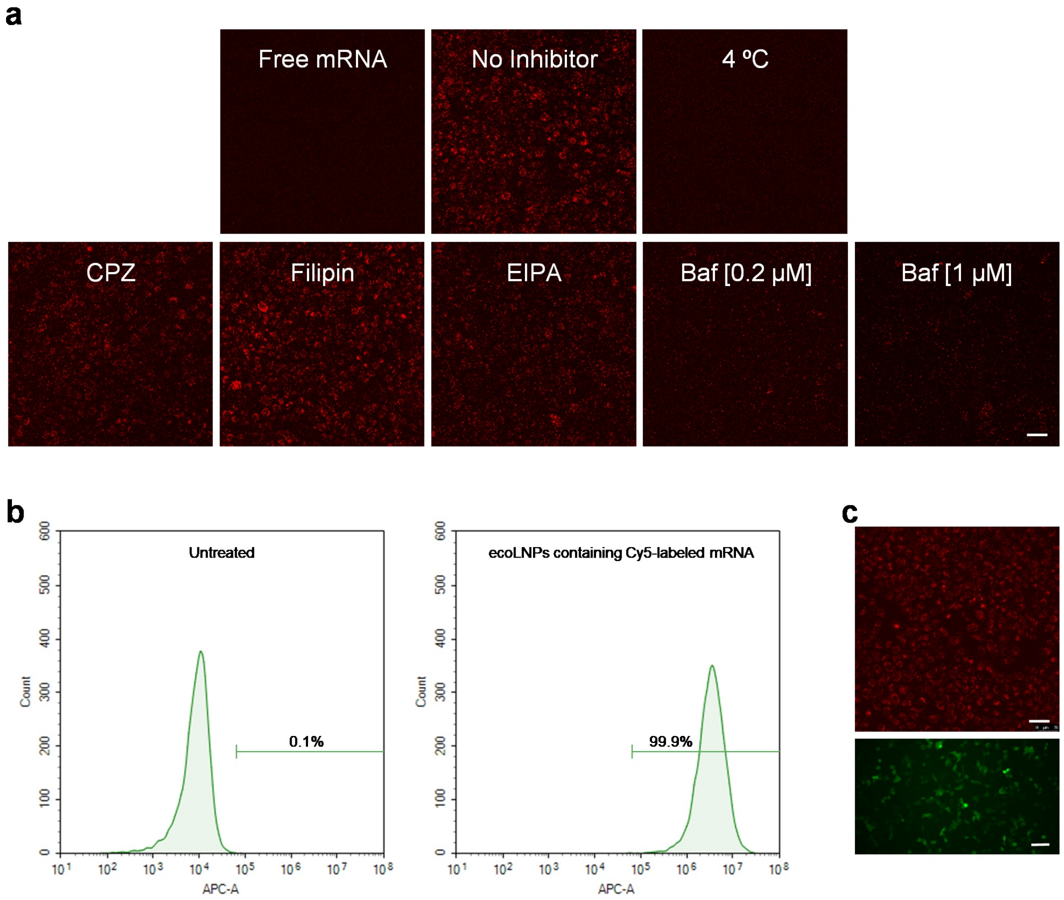

Supplementary Fig. 9 | Effect of various inhibitors and culture temperature on endocytosis of ecoLNPs as well as the cellular uptake and mRNA delivery mediated by PNT-2N6-UC18 LNPs. a Representative fluorescence microscopy images of cells after 6 h treatment with ecoLNPs containing Cy5-labeled mRNA and (or) various inhibitors at 37 °C or 4 °C. Cells treated with free Cy5-labeled mRNA were used as a negative control. Scale bar, 25 μm. **b** Cellular uptake of ecoLNPs containing Cy5-labeled mRNA determined by flow cytometry. **c** Representative fluorescence microscopy images of cells after 6 h (top panel) or 24 h (bottom panel) treatment with PNT-2N6-UC18 LNPs containing 200 ng of Cy5-labeled mRNA (top panel) or eGFP mRNA (bottom panel). Scale bar, 25 μm (top panel) and 50 μm (bottom panel).

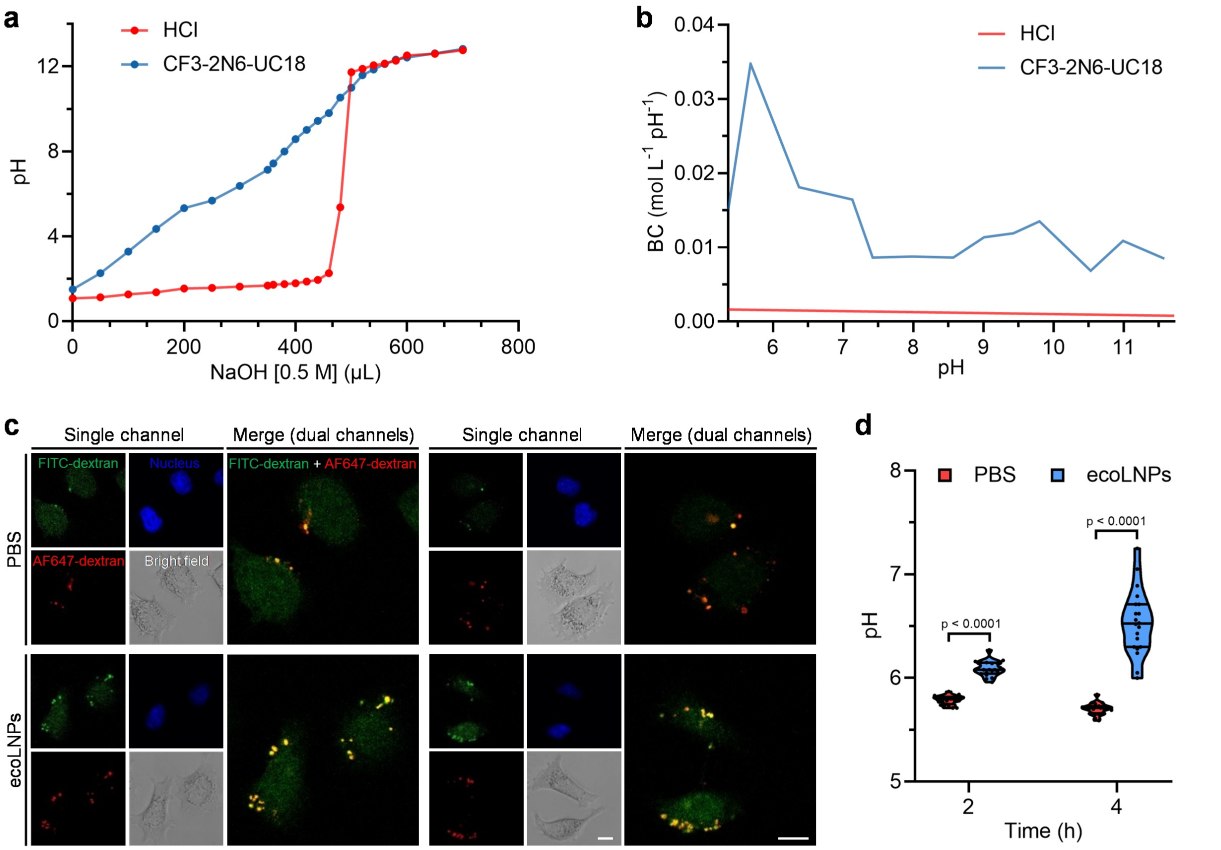

Supplementary Fig. 10 | Buffering capacity of CF3-2N6-UC18 and endo-lysosomal pH affected by ecoLNPs. a The titration curve of CF3-2N6-UC18. Aqueous HCl was included for comparison. **b** The buffer capacity of CF3-2N6-UC18 calculated from its titration curve. **c** Representative confocal microscope images of HeLa cells treated with PBS or ecoLNPs (Left, 2h. Right, 4h). Scale bar, 10 µm. **d** Endo-lysosomal pH values calculated from an $I_{FITC}\ I_{AF647}^{-1}$ versus pH curve. Violin plots indicate minimum, lower quartile, median (middle line), upper quartile, and maximum (*n* = 20 cells, two-way ANOVA with Sidak's multiple comparison test).

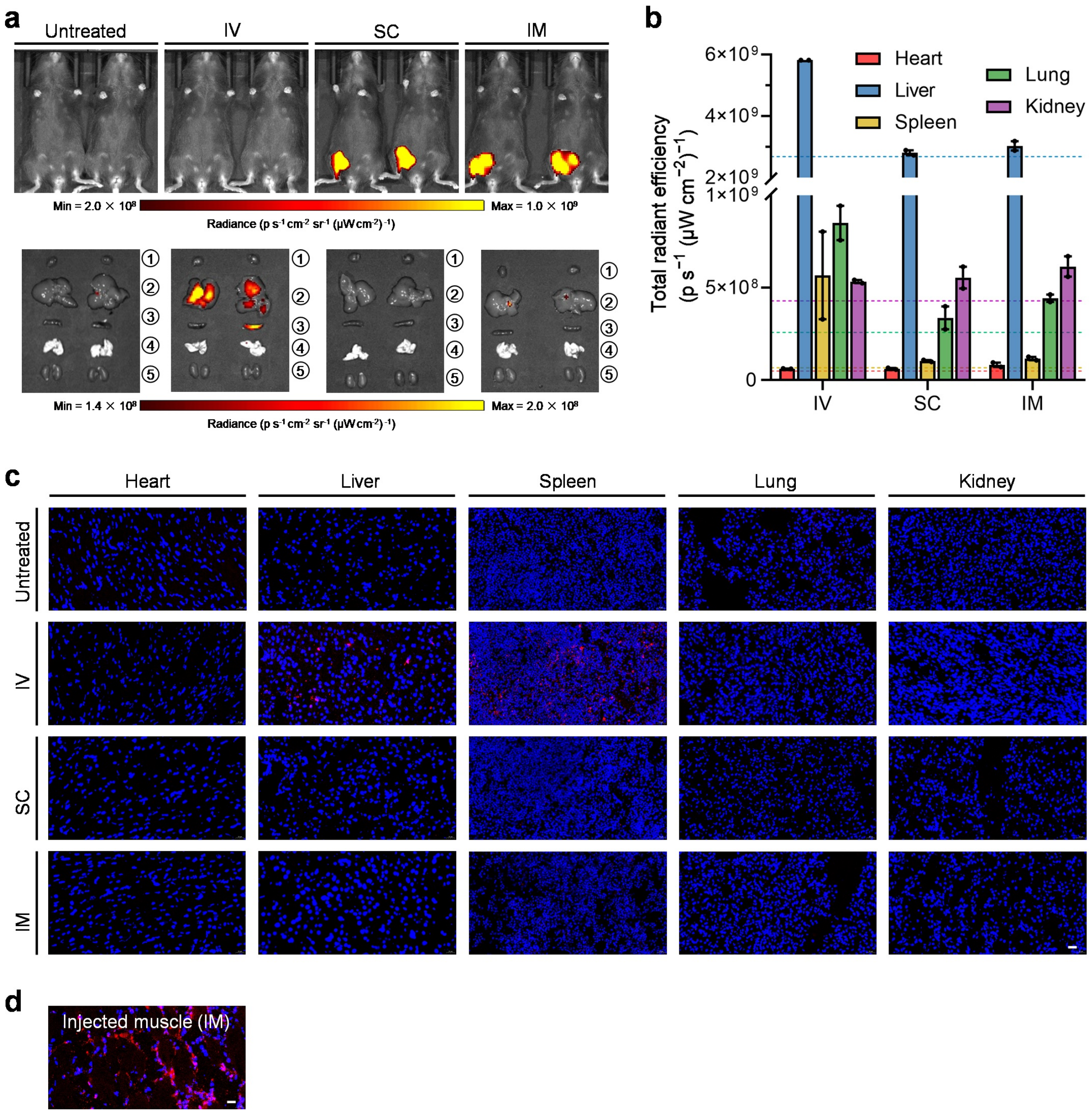

Supplementary Fig. 11 | The bio-distribution of ecoLNPs. a Representative in vivo and ex vivo fluorescence imaging of mice 4 h after a single IV, SC, or IM injection of ecoLNPs at a mRNA dose of 0.5 mg kg^{-1}. Untreated mice served as a negative control. 1, heart; 2, liver; 3, spleen; 4, lung; 5, kidney. **b** Quantification of the total radiant efficiency from major organs. Data are presented as mean ± SEM (n = 2 mice). The colored dashed lines indicated the background values of corresponding tissues from untreated mice. **c**,**d** Representative fluorescence images of frozen sections of major organs (**c**) and injected muscle (**d**). Scale bar: 20 μm.

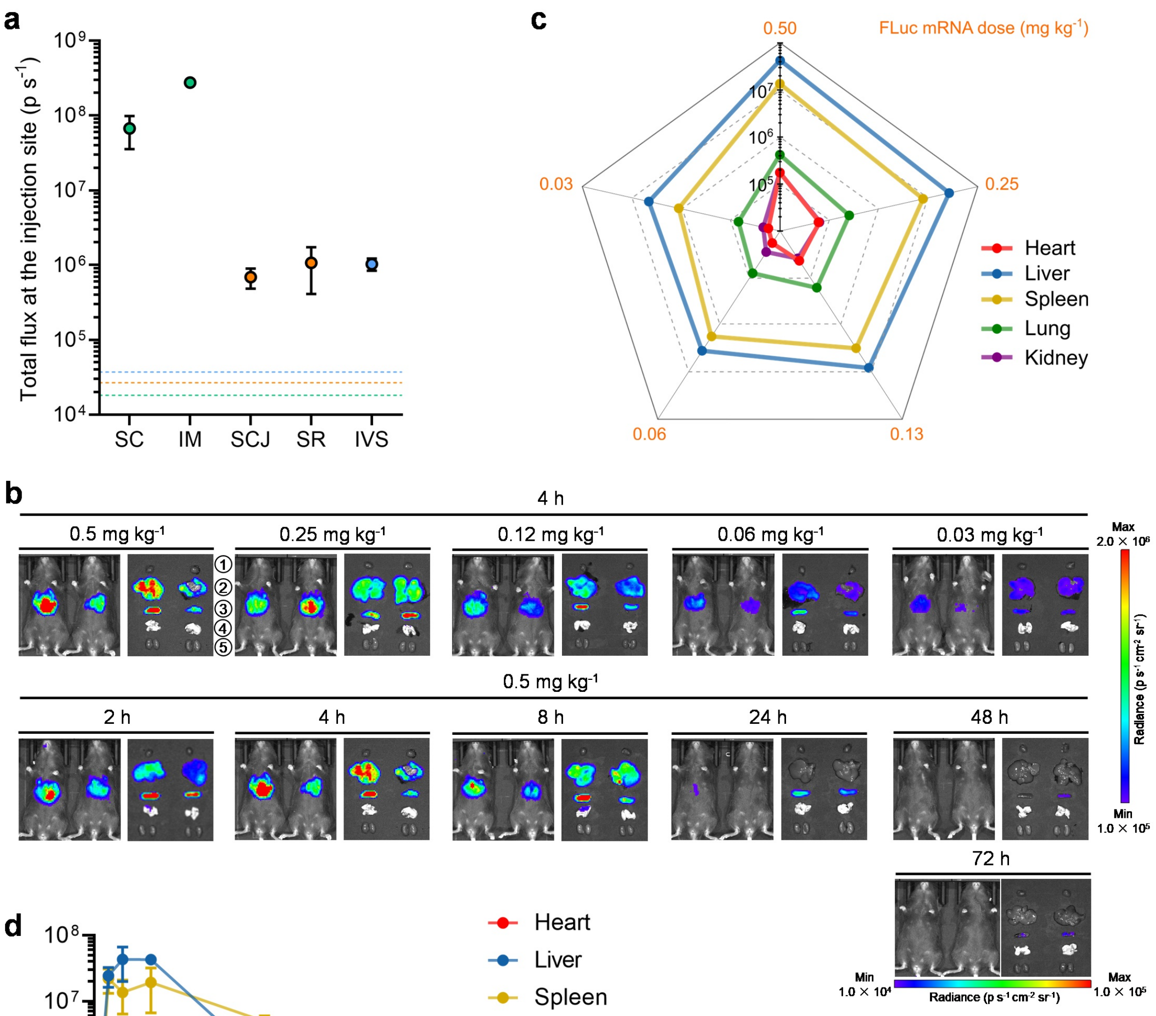

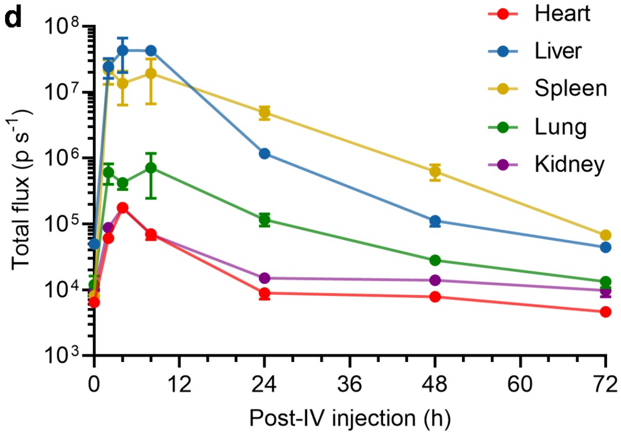

e

Organ	AUC (p s^{-1} h)
Heart	1.72×10^6
Liver	6.07×10^8
Spleen	3.71×10^8
Lung	5.49×10^6
Kidney	2.07×10^6

Supplementary Fig. 12 | Expression profiles of ecoLNPs-encapsulated mRNA. a Quantification of the total flux (p s^{-1}) at the injection sites 4 h after local delivery of ecoLNPs-encapsulated FLuc mRNA (see Fig. 6a). The dashed lines indicated the corresponding local background values from untreated mice. Data are presented as mean ± SEM. **b** In vivo and ex vivo bioluminescence imaging of mice 4 h after a single IV injection of ecoLNPs at varying mRNA

doses (0.50, 0.25, 0.12, 0.06, or 0.03 mg kg^{-1}) (top panel) or 2, 4, 8, 24, 48, or 72 h after a single IV injection of ecoLNPs (equivalent to 0.5 mg kg^{-1} of mRNA) (bottom panel). Both panels shared the 0.5 mg kg^{-1} & 4h-treated group from Fig. 6a to show the expression profile of ecoLNPs-encapsulated FLuc mRNA. 1, heart; 2, liver; 3, spleen; 4, lung; 5, kidney. **c**,**d** Quantification of the total flux from the major organs in **b**. Data are presented as mean ± SEM (*n* = 2 mice). Data at 0 h in d indicated the background value of each organ examined. **e** Area under curve (AUC) of major organs in **d**.

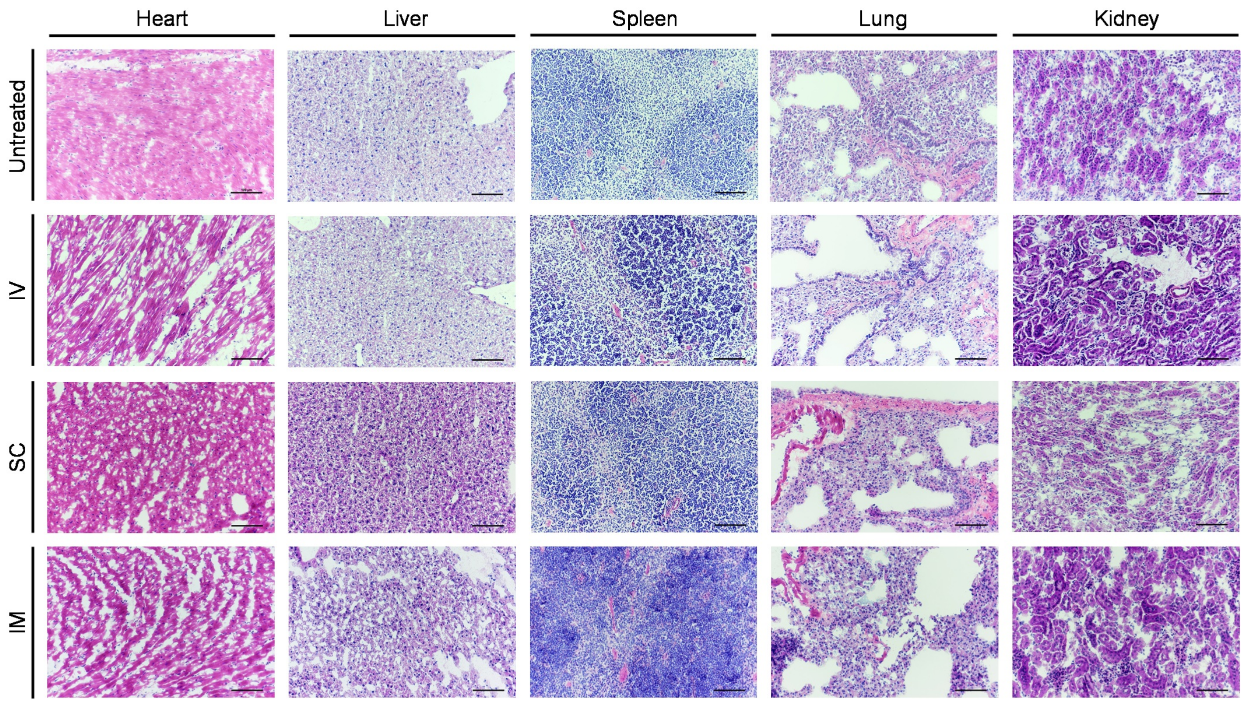

Supplementary Fig. 13 | In vivo safety evaluation of ecoLNPs. Hematoxylin- and eosin-stained histologic sections of major tissues from mice 4 h after IV, SC, and IM injection of ecoLNPs at a mRNA dose of 0.5 mg kg^{-1}. Histologic sections from untreated mice served as a control group. Scale bar, 100 μm.

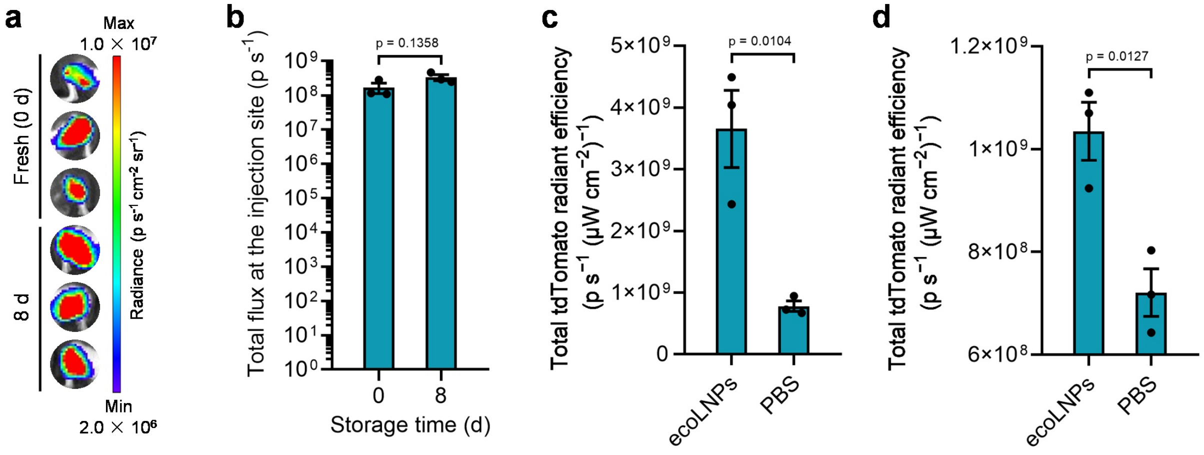

Supplementary Fig. 14 | In vivo delivery performance of ecoLNPs after 8 days of storage and in vivo genome editing mediated by ecoLNPs. a In vivo bioluminescence imaging of mice 4 h after a single IM injection of fresh ecoLNPs (0 d) or ecoLNPs stored at 4 °C for 8 d at a mRNA dose of 0.5 mg kg^{-1}. **b** Quantification of the total flux (p s^{-1}) at the injection sites from **a**. (n = 3 legs, unpaired, two-tailed Student's t-test). **c**,**d** Quantification of tdTomato fluorescence intensity of ex vivo tissues in Fig. 6g (**c**) and 6h (**d**) (n =3, unpaired, two-tailed Student's t-test). For all relevant panels, data are presented as mean ± SEM.

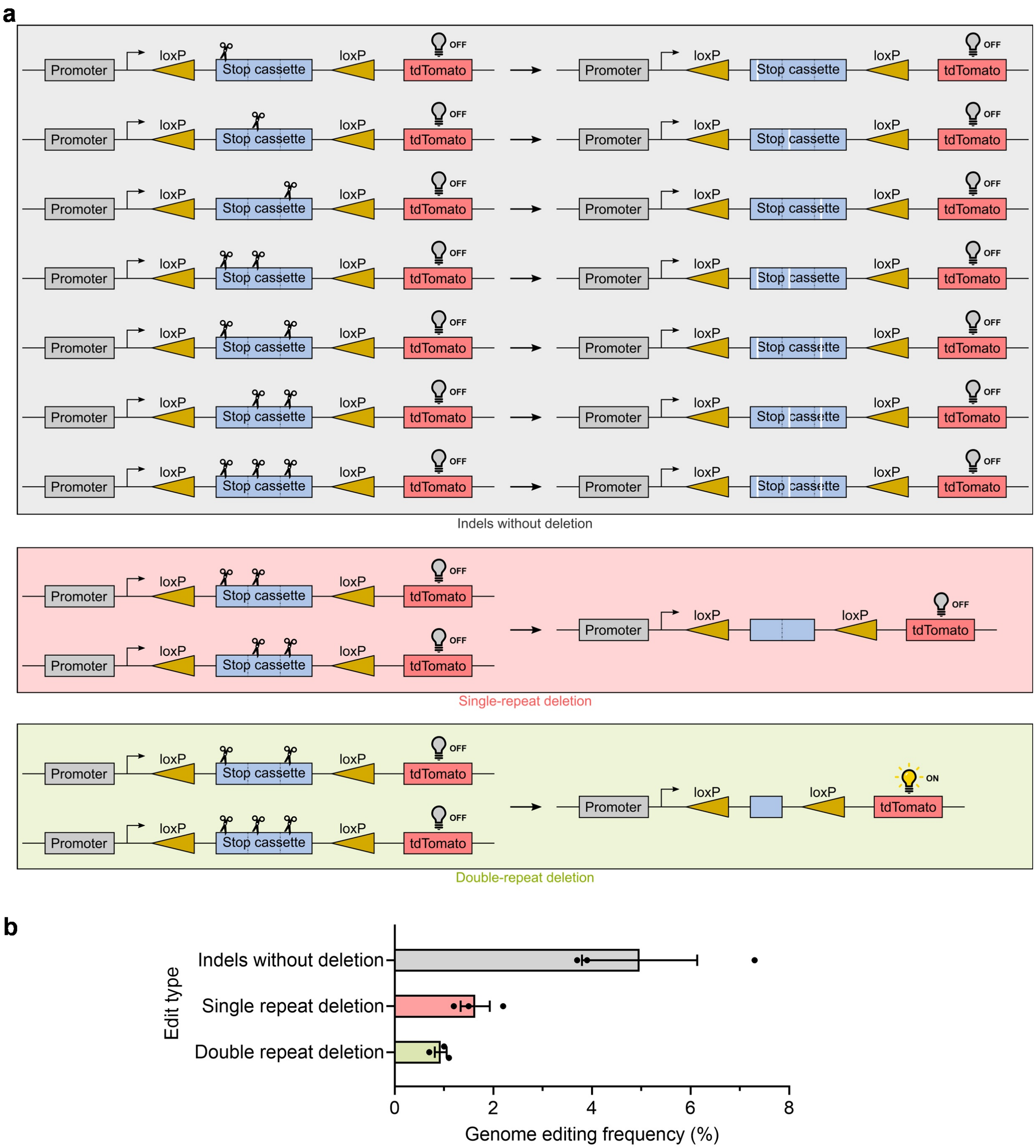

Supplementary Fig. 15 | The frequency of genome editing in the injected tissues determined by third-generation sequencing. a,**b** Edit types (**a**) and their genome editing frequencies (**b**) in the injection sites were confirmed by third-generation sequencing. Data are presented as mean ± SEM (n = 3 legs).

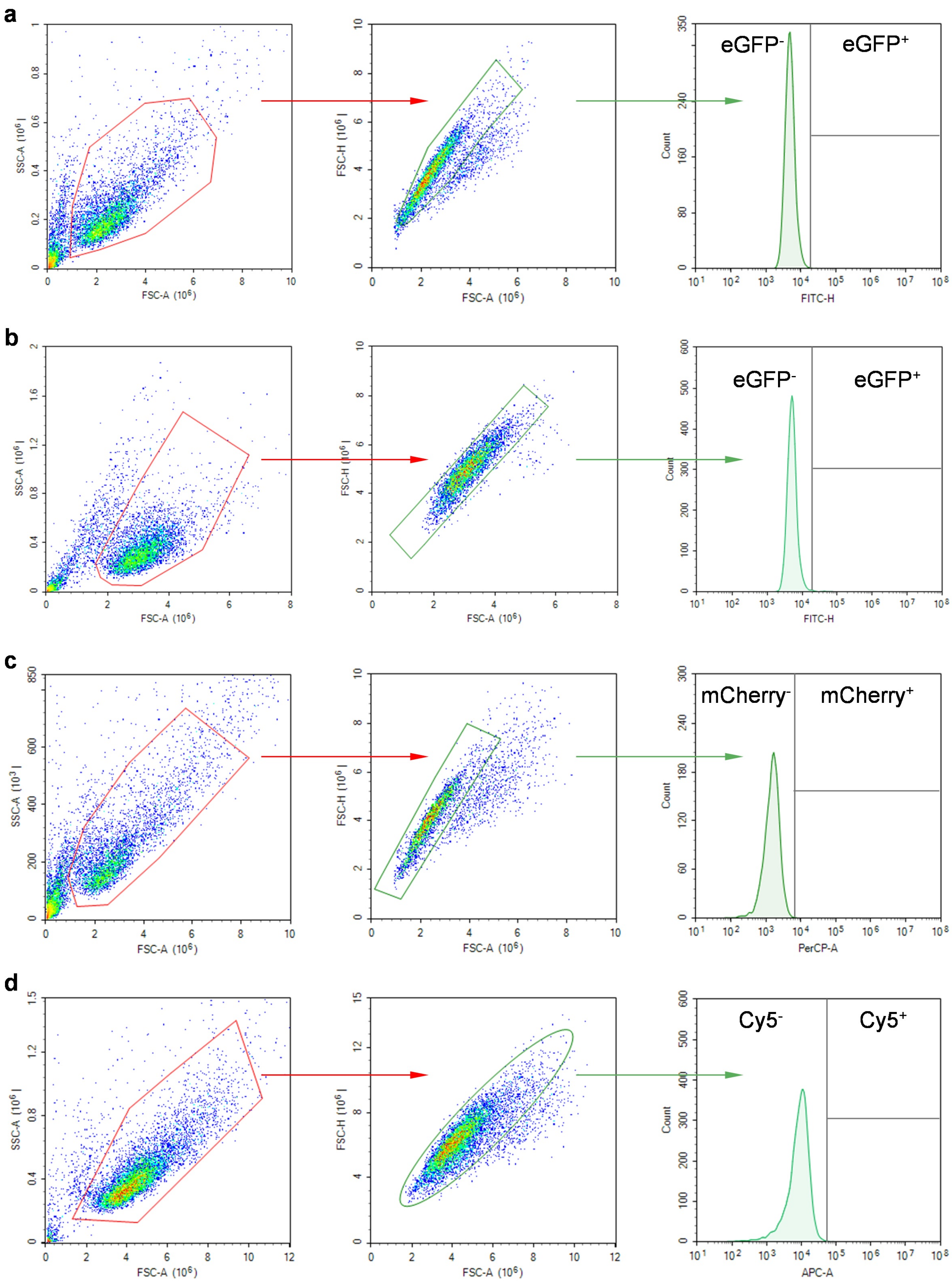

a
b
c
d
SSC-A (10⁶)
FSC-A (10⁶)
FSC-H (10⁶)
SSC-A (10³)
Count
eGFP-
eGFP+
mCherry-
mCherry+
Cy5-
Cy5+
FITC-H
PerCP-A
APC-A

Supplementary Fig. 16 | Gating strategies for analysis of GFP-, mCherry-, and Cy5-positive cells. a Gating strategy for Figs. 4e, 4f (left panel) and Supplementary Fig. 8e. **b** Gating strategy in Fig. 4f (right panel); **c** Gating strategy for Fig. 4h (right panel). **d** Gating strategy for Fig. 5a and Supplementary Fig. 9b.

Supplementary Table 1 | The basic information of chloroquine-like lipids used in this study.

No.	Compound	IUPAC name	Formula	MW (g/mol)	*m/z* (M+H)[+]	tPSA	clog P
1	CF3-3N6-C8	*N*[1],*N*[1],*N*[6]-trioctyl-*N*[6]-(6-((7-(trifluoromethyl)quinolin-4-yl)amino)hexyl)hexane-1,6-diamine	$C_{46}H_{81}F_3N_4$	747.2	747.6122<br>374.3123[a]	30.87	17.54
2	CF3-3N6-C9	*N*[1],*N*[1],*N*[6]-trinonyl-*N*[6]-(6-((7-(trifluoromethyl)quinolin-4-yl)amino)hexyl)hexane-1,6-diamine	$C_{49}H_{87}F_3N_4$	789.3	789.6526<br>395.3337[a]	30.87	19.13
3	CF3-3N6-C10	*N*[1],*N*[1],*N*[6]-tris(decyl)-*N*[6]-(6-((7-(trifluoromethyl)quinolin-4-yl)amino)hexyl)hexane-1,6-diamine	$C_{52}H_{93}F_3N_4$	831.3	831.7379<br>416.3718[a]	30.87	20.72
4	CF3-3N6-C11	*N*[1]-(6-((7-(trifluoromethyl)quinolin-4-yl)amino)hexyl)-*N*[1],*N*[6],*N*[6]-triundecylhexane-1,6-diamine	$C_{55}H_{99}F_3N_4$	873.4	873.7458<br>437.3787[a]	30.87	22.31
5	CF3-3N6-C12	*N*[1],*N*[1],*N*[6]-tridodecyl-*N*[6]-(6-((7-(trifluoromethyl)quinolin-4-yl)amino)hexyl)hexane-1,6-diamine	$C_{58}H_{105}F_3N_4$	915.5	915.8504<br>458.4305[a]	30.87	23.89
6	CF3-3N6-C13	*N*[1],*N*[1],*N*[6]-tris(tridecyl)-*N*[6]-(6-((7-(trifluoromethyl)quinolin-4-yl)amino)hexyl)hexane-1,6-diamine	$C_{61}H_{111}F_3N_4$	957.6	957.8396<br>479.4287[a]	30.87	25.48
7	CF3-3N6-C14	*N*[1],*N*[1],*N*[6]-tritetradecyl-*N*[6]-(6-((7-(trifluoromethyl)quinolin-4-yl)amino)hexyl)hexane-1,6-diamine	$C_{64}H_{117}F_3N_4$	999.7	999.9261<br>500.4662[a]	30.87	27.07
8	CF3-3N6-C15	*N*[1],*N*[1],*N*[6]-tripentadecyl-*N*[6]-(6-((7-(trifluoromethyl)quinolin-4-yl)amino)hexyl)hexane-1,6-diamine	$C_{67}H_{123}F_3N_4$	1041.7	1041.9415<br>521.4773[a]	30.87	28.65
9	CF3-3N6-C16	*N*[1],*N*[1],*N*[6]-trihexadecyl-*N*[6]-(6-((7-(trifluoromethyl)quinolin-4-yl)amino)hexyl)hexane-1,6-diamine	$C_{70}H_{129}F_3N_4$	1083.8	542.4943[a]	30.87	30.24
10	CF3-3N6-C17	*N*[1],*N*[1],*N*[6]-triheptadecyl-*N*[6]-(6-((7-(trifluoromethyl)quinolin-4-yl)amino)hexyl)hexane-1,6-diamine	$C_{73}H_{135}F_3N_4$	1125.9	1126.0673<br>563.5368[a]	30.87	31.83

11	CF3-3N6-C18	*N*[1],*N*[1],*N*[6]-trioctadecyl-*N*[6]-(6-((7-(trifluoromethyl)quinolin-4-yl)amino)hexyl)hexane-1,6-diamine	$C_{76}H_{141}F_3N_4$	1168.0	584.5405[a]	30.87	33.41
12	CF3-3N6-UC18	*N*[1],*N*[1],*N*[6]-tri((Z)-octadec-9-en-1-yl)-*N*[6]-(6-((7-(trifluoromethyl)quinolin-4-yl)amino)hexyl)hexane-1,6-diamine	$C_{76}H_{135}F_3N_4$	1161.9	1161.9140<br>581.4582[a]	30.87	31.96
13	CF3-3N6-C22	*N*[1],*N*[1],*N*[6]-tridocosyl-*N*[6]-(6-((7-(trifluoromethyl)quinolin-4-yl)amino)hexyl)hexane-1,6-diamine	$C_{88}H_{165}F_3N_4$	1336.3	1337.3114<br>669.1558[a]	30.87	39.76
14	F-3N6-C12	*N*[1],*N*[1],*N*[6]-tridodecyl-*N*[6]-(6-((7-fluoroquinolin-4-yl)amino)hexyl)hexane-1,6-diamine	$C_{57}H_{105}FN_4$	865.5	865.8497	30.87	23.08
15	F-3N6-C17	*N*[1]-(6-((7-fluoroquinolin-4-yl)amino)hexyl)-*N*[1],*N*[6],*N*[6]-triheptadecylhexane-1,6-diamine	$C_{72}H_{135}FN_4$	1075.9	1076.0897	30.87	31.01
16	F-3N6-UC18	*N*[1]-(6-((7-fluoroquinolin-4-yl)amino)hexyl)-*N*[1],*N*[6],*N*[6]-tri((Z)-octadec-9-en-1-yl)hexane-1,6-diamine	$C_{75}H_{135}FN_4$	1111.9	1112.0895<br>556.5467[a]	30.87	31.15
17	Cl-3N6-C12	*N*[1]-(6-((7-chloroquinolin-4-yl)amino)hexyl)-*N*[1],*N*[6],*N*[6]-tridodecylhexane-1,6-diamine	$C_{57}H_{105}ClN_4$	881.9	881.8167<br>441.4125[a]	30.87	23.65
18	Cl-3N6-C17	*N*[1]-(6-((7-chloroquinolin-4-yl)amino)hexyl)-*N*[1],*N*[6],*N*[6]-triheptadecylhexane-1,6-diamine	$C_{72}H_{135}ClN_4$	1092.4	1092.0602<br>546.5321[a]	30.87	31.58
19	Cl-3N6-UC18	*N*[1]-(6-((7-chloroquinolin-4-yl)amino)hexyl)-*N*[1],*N*[6],*N*[6]-tri((Z)-octadec-9-en-1-yl)hexane-1,6-diamine	$C_{75}H_{135}ClN_4$	1128.4	564.0189[a]	30.87	31.72
20	Br-3N6-C12	*N*[1]-(6-((7-bromoquinolin-4-yl)amino)hexyl)-*N*[1],*N*[6],*N*[6]-tridodecylhexane-1,6-diamine	$C_{57}H_{105}BrN_4$	926.4	927.7717<br>464.3882[a]	30.87	23.80
21	Br-3N6-C17	*N*[1]-(6-((7-bromoquinolin-4-yl)amino)hexyl)-*N*[1],*N*[6],*N*[6]-triheptadecylhexane-1,6-diamine	$C_{72}H_{135}BrN_4$	1136.8	569.5075[a]	30.87	31.73

22	Br-3N6-UC18	*N*[1]-(6-((7-bromoquinolin-4-yl)amino)hexyl)-*N*[1],*N*[6],*N*[6]-tri((Z)-octadec-9-en-1-yl)hexane-1,6-diamine	$C_{75}H_{135}BrN_4$	1172.8	1171.9796	30.87	31.87
23	I-3N6-C12	*N*[1],*N*[1],*N*[6]-tridodecyl-*N*[6]-(6-((7-iodoquinolin-4-yl)amino)hexyl)hexane-1,6-diamine	$C_{57}H_{105}IN_4$	973.4	973.7888<br>487.4356[a]	30.87	24.06
24	I-3N6-C17	*N*[1],*N*[1],*N*[6]-triheptadecyl-*N*[6]-(6-((7-iodoquinolin-4-yl)amino)hexyl)hexane-1,6-diamine	$C_{72}H_{135}IN_4$	1183.8	1183.9993<br>592.5011[a]	30.87	31.99
25	I-3N6-UC18	*N*[1]-(6-((7-iodoquinolin-4-yl)amino)hexyl)-*N*[1],*N*[6],*N*[6]-tri((Z)-octadec-9-en-1-yl)hexane-1,6-diamine	$C_{75}H_{135}IN_4$	1219.8	1220.0007<br>610.5009[a]	30.87	32.13
26	MeO-3N6-C12	N*[1]*,*N*[1],*N*[6]-tridodecyl-*N*[6]-(6-((7-methoxyquinolin-4-yl)amino)hexyl)hexane-1,6-diamine	$C_{58}H_{108}N_4O$	877.5	439.4390[a]	40.1	23.15
27	MeO-3N6-C17	*N*[1],*N*[1],*N*[6]-triheptadecyl-*N*[6]-(6-((7-methoxyquinolin-4-yl)amino)hexyl)hexane-1,6-diamine	$C_{73}H_{138}N_4O$	1087.9	1088.1759	40.1	31.09
28	MeO-3N6-UC18	*N*[1]-(6-((7-methoxyquinolin-4-yl)amino)hexyl)-*N*[1],*N*[6],*N*[6]-tri((Z)-octadec-9-en-1-yl)hexane-1,6-diamine	$C_{76}H_{138}N_4O$	1124.0	562.5577[a]	40.1	31.22
29	2MeO-3N6-C12	*N*[1]-(6-((6,7-dimethoxyquinolin-4-yl)amino)hexyl)-*N*[1],*N*[6],*N*[6]-tridodecylhexane-1,6-diamine	$C_{59}H_{110}N_4O_2$	907.6	907.9985	49.33	23.09
30	2MeO-3N6-C17	*N*[1]-(6-((6,7-dimethoxyquinolin-4-yl)amino)hexyl)-*N*[1],*N*[6],*N*[6]-triheptadecylhexane-1,6-diamine	$C_{74}H_{140}N_4O_2$	1118.0	559.1627[a]	49.33	31.02
31	2MeO-3N6-UC18	*N*[1]-(6-((6,7-dimethoxyquinolin-4-yl)amino)hexyl)-*N*[1],*N*[6],*N*[6]-tri((Z)-octadec-9-en-1-yl)hexane-1,6-diamine	$C_{77}H_{140}N_4O_2$	1154.0	1154.1244<br>577.5635[a]	49.33	31.16
32	PNT-3N6-C12	*N*[1]-(6-((1,10-phenanthrolin-4-yl)amino)hexyl)-*N*[1],*N*[6],*N*[6]-tridodecylhexane-1,6-diamine	$C_{60}H_{107}N_5$	898.6	449.9331[a]	43.23	22.94

33	PNT-3N6-C17	N^1-(6-((1,10-phenanthrolin-4-yl)amino)hexyl)-N^1,N^6,N^6-triheptadecylhexane-1,6-diamine	$C_{75}H_{137}N_5$	1109.0	555.0518[a]	43.23	30.88
34	PNT-3N6-UC18	N^1-(6-((1,10-phenanthrolin-4-yl)amino)hexyl)-N^1,N^6,N^6-tri((Z)-octadec-9-en-1-yl)hexane-1,6-diamine	$C_{78}H_{137}N_5$	1145.0	1145.1031<br>573.0528[a]	43.23	31.01
35	AD-3N6-C12	N^1-(6-((6-chloro-2-methoxyacridin-9-yl)amino)hexyl)-N^1,N^6,N^6-tridodecylhexane-1,6-diamine	$C_{62}H_{109}ClN_4O$	962.0	961.8467	40.10	25.31
36	AD-3N6-C17	N^1-(6-((6-chloro-2-methoxyacridin-9-yl)amino)hexyl)-N^1,N^6,N^6-triheptadecylhexane-1,6-diamine	$C_{77}H_{139}ClN_4O$	1172.4	586.5296[a]	40.10	33.25
37	AD-3N6-UC18	N^1-(6-((6-chloro-2-methoxyacridin-9-yl)amino)hexyl)-N^1,N^6,N^6-tri((Z)-octadec-9-en-1-yl)hexane-1,6-diamine	$C_{80}H_{139}ClN_4O$	1208.5	604.5448[a]	40.10	33.38
38	CF3-2N4-UC18	N^1,N^1-di((Z)-octadec-9-en-1-yl)-N^4-(7-(trifluoromethyl)quinolin-4-yl)butane-1,4-diamine	$C_{50}H_{84}F_3N_3$	784.2	784.6671<br>392.8377[a]	27.63	20.96
39	CF3-2N6-UC18	N^1,N^1-di((Z)-octadec-9-en-1-yl)-N^6-(7-(trifluoromethyl)quinolin-4-yl)hexane-1,6-diamine	$C_{52}H_{88}F_3N_3$	812.3	812.6930<br>406.8525[a]	27.63	22.01
40	CF3-3N2-C12	N^1,N^1,N^2-tridodecyl-N^2-(2-((7-(trifluoromethyl)quinolin-4-yl)amino)ethyl)ethane-1,2-diamine	$C_{50}H_{89}F_3N_4$	803.3	803.7182<br>402.3633[a]	30.87	21.78
41	CF3-3N2-C17	N^1,N^1,N^2-triheptadecyl-N^2-(2-((7-(trifluoromethyl)quinolin-4-yl)amino)ethyl)ethane-1,2-diamine	$C_{65}H_{119}F_3N_4$	1013.7	1014.7051<br>507.8587[a]	30.87	29.71
42	CF3-3N2-UC18	N^1,N^1,N^2-tri((Z)-octadec-9-en-1-yl)-N^2-(2-((7-(trifluoromethyl)quinolin-4-yl)amino)ethyl)ethane-1,2-diamine	$C_{68}H_{119}F_3N_4$	1049.7	1049.9529<br>525.4812[a]	30.87	29.85
43	CF3-SPN-C12	N^1-(3-(didodecylamino)propyl)-N^1,N^4-didodecyl-N^4-(3-((7-(trifluoromethyl)quinolin-4-yl)amino)propyl)butane-1,4-diamine	$C_{68}H_{126}F_3N_5$	1070.8	536.0104[a]	34.11	28.55

44	CF3-SPN-C17	*N*[1]-(3-(diheptadecylamino)propyl)-*N*[1],*N*[4]-diheptadecyl-*N*[4]-(3-((7-(trifluoromethyl)quinolin-4-yl)amino)propyl)butane-1,4-diamine	$C_{88}H_{166}F_{3}N_{5}$	1351.3	1351.3264	34.11	39.13
45	CF3-SPN-UC18	*N*[1]-(3-(di((Z)-octadec-9-en-1-yl)amino)propyl)-*N*[1],*N*[4]-di((Z)-octadec-9-en-1-yl)-*N*[4]-(3-((7-(trifluoromethyl)quinolin-4-yl)amino)propyl)butane-1,4-diamine	$C_{92}H_{166}F_{3}N_{5}$	1399.4	700.6693[a]	34.11	39.31
46	PNT-2N6-UC18[b]	*N*[1],*N*[1]-di((Z)-octadec-9-en-1-yl)-*N*[6]-(1,10-phenanthrolin-4-yl)hexane-1,6-diamine	$C_{54}H_{9}0N_{4}$	795.3	795.7318<br>398.3720[a]	39.99	21.07

[a]*m/z* $(M+2H)^{2+}$.

[b]A control lipid used for the endosomal escape assay.

Supplementary Table 2 | The first-round 4^5 CCD applied to CF3-3N6-C12 formulation optimization.

Formulation No.	Ethanol phase (molar percentage)				CF3-3N6-C12:mRNA (weight ratio)
	CF3-3N6-C12	DOPE	Cholesterol	DMG-PEG2k	
1st-F1	22	33.1	44.1	0.8	10
1st-F2	28.3	42.4	28.2	1.1	10
1st-F3	18	36	45	1	6
1st-F4	22.1	44.2	33.3	0.4	14
1st-F5	21.9	32.8	43.7	1.6	10
1st-F6	18.1	36.2	45.3	0.4	14
1st-F7	18.1	27.1	54.1	0.7	10
1st-F8	22.1	22.1	55.4	0.4	6
1st-F9	28.4	28.4	42.6	0.6	14
1st-F10	22	22	54.8	1.2	6
1st-F11	18	36	45	1	14
1st-F12	22	43.9	32.9	1.2	14
1st-F13	28.1	28.1	42.3	1.5	6
1st-F14	28.4	28.4	42.6	0.6	6
1st-F15	22	43.9	32.9	1.2	6
1st-F16	28.3	14.1	56.5	1.1	10
1st-F17	18.1	45.1	36.1	0.7	10
1st-F18	28.1	28.1	42.3	1.5	14
1st-F19	18.1	36.2	45.3	0.4	6
1st-F20	22	22	54.8	1.2	14
1st-F21	22.1	44.2	33.3	0.4	6
1st-F22	22	33.1	44.1	0.8	2
1st-F23	22.2	33.3	44.4	0.1	10
1st-F24	22	33.1	44.1	0.8	18
1st-F25	22.1	22.1	55.4	0.4	14

Supplementary Table 3 | The second-round 4^5 CCD applied to CF3-3N6-C12 formulation optimization.

Formulation No.	Ethanol phase (molar percentage)				CF3-3N6-C12:mRNA (weight ratio)
	CF3-3N6-C12	DOPE	Cholesterol	DMG-PEG2k	
2nd-F1	18.1	27.2	54.4	0.3	4
2nd-F2	15.3	23	61.5	0.2	4
2nd-F3	18.1	18.1	63.6	0.2	5
2nd-F4	15.3	30.7	53.7	0.3	3
2nd-F5	18.1	18.1	63.5	0.3	3
2nd-F6	22.2	22.2	55.3	0.3	5
2nd-F7	22.1	22.1	55.4	0.4	3
2nd-F8	18.1	36.2	45.4	0.3	3
2nd-F9	15.4	30.7	53.7	0.2	5
2nd-F10	18.1	27.2	54.3	0.4	4
2nd-F11	18.1	18.1	63.6	0.2	3
2nd-F12	22.2	22.2	55.3	0.3	3
2nd-F13	18.1	18.1	63.5	0.3	5
2nd-F14	15.3	38.4	46.1	0.2	4
2nd-F15	18.1	27.2	54.4	0.3	2
2nd-F16	22.1	11.1	66.5	0.3	4
2nd-F17	18.1	36.3	45.4	0.2	3
2nd-F18	15.4	30.7	53.7	0.2	3
2nd-F19	22.1	22.1	55.4	0.4	5
2nd-F20	18.1	36.2	45.4	0.3	5
2nd-F21	15.3	30.7	53.7	0.3	5
2nd-F22	18.1	27.2	54.5	0.2	4
2nd-F23	18.1	36.3	45.4	0.2	5
2nd-F24	22.1	33.2	44.4	0.3	4
2nd-F25	18.1	27.2	54.4	0.3	6

Supplementary Table 4 | The third-round 4^5 CCD applied to CF3-3N6-C17 formulation optimization.

Formulation No.	Ethanol phase (molar percentage)				CF3-3N6-C17:mRNA (weight ratio)
	CF3-3N6-C17	DOPE	Cholesterol	DMG-PEG2k	
3rd-F1	18.1	27.2	54.4	0.3	4
3rd-F2	15.3	23	61.5	0.2	4
3rd-F3	18.1	18.1	63.6	0.2	5
3rd-F4	15.3	30.7	53.7	0.3	3
3rd-F5	18.1	18.1	63.5	0.3	3
3rd-F6	22.2	22.2	55.3	0.3	5
3rd-F7	22.1	22.1	55.4	0.4	3
3rd-F8	18.1	36.2	45.4	0.3	3
3rd-F9	15.4	30.7	53.7	0.2	5
3rd-F10	18.1	27.2	54.3	0.4	4
3rd-F11	18.1	18.1	63.6	0.2	3
3rd-F12	22.2	22.2	55.3	0.3	3
3rd-F13	18.1	18.1	63.5	0.3	5
3rd-F14	15.3	38.4	46.1	0.2	4
3rd-F15	18.1	27.2	54.4	0.3	2
3rd-F16	22.1	11.1	66.5	0.3	4
3rd-F17	18.1	36.3	45.4	0.2	3
3rd-F18	15.4	30.7	53.7	0.2	3
3rd-F19	22.1	22.1	55.4	0.4	5
3rd-F20	18.1	36.2	45.4	0.3	5
3rd-F21	15.3	30.7	53.7	0.3	5
3rd-F22	18.1	27.2	54.5	0.2	4
3rd-F23	18.1	36.3	45.4	0.2	5
3rd-F24	22.1	33.2	44.4	0.3	4
3rd-F25	18.1	27.2	54.4	0.3	6

Supplementary Table 5 | Serum biochemistry parameters of mice 72 h after treatment with ecoLNPs.

Parameter	Unit	ecoLNPs	PBS
Albumin	g l^{-1}	28.65 ± 0.92	28.5 ± 1.98[a]
Triglyceride	mmol l^{-1}	1.33 ± 0.27	0.96 ± 0.21
Inorganic phosphorus	mmol l^{-1}	3.025 ± 0.4	2.609 ± 0.27
Glucose	mmol l^{-1}	16.385 ± 1.66	17.42 ± 0.93
Total protein	g l^{-1}	45.2 ± 1.84	45.3 ± 2.83
Alanine aminotransferase	U l^{-1}	34.255 ± 1.61	23.49 ± 8.73
aspartic transaminase	U l^{-1}	218.225 ± 124.73	119.005 ± 0.29
calcium	mmol l^{-1}	2.375 ± 0.08	2.425 ± 0.09
Amylase	U l^{-1}	2318.5 ± 187.38	2471 ± 264.46
Creatine kinase	U l^{-1}	1173.15 ± 356.31	533.25 ± 84.78
Albumin Globulin Ratio	A G^{-1}	1.73 ± 0.04	1.695 ± 0.04
Urinary protein creatinine ratio	B C^{-1}	679.655 ± 143.83	621.02 ± 73.78
Globulin	g l^{-1}	16.55 ± 0.92	16.8 ± 0.85
Crea	μmol l^{-1}	12.25 ± 0.64	13 ± 1.13
blood urea nitrogen	mmol l^{-1}	8.28 ± 1.33	8.115 ± 1.66

Data were presented as mean ± SEM. Mice treated with PBS were used for comparison (n = 2, unpaired, two-tailed Student's t-test). No statistically significant difference was observed for all parameters tested.
