# Supplementary material for: Structure-guided design of endosomolytic chloroquine-like lipid nanoparticles for mRNA delivery and genome editing
Source: Nat Commun. 2025 May 7;16:4241. doi: 10.1038/s41467-025-59501-y (PMC12058976; doi:10.1038/s41467-025-59501-y)
Supplement: Supplementary file 2 — Reporting Summary [file 41467_2025_59501_MOESM2_ESM.pdf]

nature portfolio

Corresponding author(s): Bin Li

Last updated by author(s): Apr 18, 2025

Reporting Summary

Nature Portfolio wishes to improve the reproducibility of the work that we publish. This form provides structure for consistency and transparency in reporting. For further information on Nature Portfolio policies, see our Editorial Policies and the Editorial Policy Checklist.

Statistics

For all statistical analyses, confirm that the following items are present in the figure legend, table legend, main text, or Methods section.

n/a	Confirmed	
☐	☒	The exact sample size (*n*) for each experimental group/condition, given as a discrete number and unit of measurement
☐	☒	A statement on whether measurements were taken from distinct samples or whether the same sample was measured repeatedly
☐	☒	The statistical test(s) used AND whether they are one- or two-sided<br>*Only common tests should be described solely by name; describe more complex techniques in the Methods section.*
☒	☐	A description of all covariates tested
☒	☐	A description of any assumptions or corrections, such as tests of normality and adjustment for multiple comparisons
☐	☒	A full description of the statistical parameters including central tendency (e.g. means) or other basic estimates (e.g. regression coefficient) AND variation (e.g. standard deviation) or associated estimates of uncertainty (e.g. confidence intervals)
☐	☒	For null hypothesis testing, the test statistic (e.g. *F*, *t*, *r*) with confidence intervals, effect sizes, degrees of freedom and *P* value noted<br>*Give P values as exact values whenever suitable.*
☒	☐	For Bayesian analysis, information on the choice of priors and Markov chain Monte Carlo settings
☒	☐	For hierarchical and complex designs, identification of the appropriate level for tests and full reporting of outcomes
☒	☐	Estimates of effect sizes (e.g. Cohen's *d*, Pearson's *r*), indicating how they were calculated

Our web collection on statistics for biologists contains articles on many of the points above.

Software and code

Policy information about availability of computer code

Data collection	Particle Solution (version 3.6.0.7122) for particle size and zeta potential measurements;<br>Gen5 software (version 3.08) for in vitro luminescence readings;<br>NovoExpress software (Version 1.6.1) for flow cytometry;<br>ZEN 3.1 (version 3.1.0.00003) for in vitro fluorescence imaging;<br>Leica Application Suite X (version 3.5.6.21594) for confocal fluorescence imaging;<br>Living Image software (version 4.4) for in vivo luminescence and fluorescence imaging;<br>AutoDock Vina (version 1.1.2) for molecular docking.
Data analysis	GraphPad Prism (version 9.5.1);<br>PyMOL (version 2.6.0a0).

For manuscripts utilizing custom algorithms or software that are central to the research but not yet described in published literature, software must be made available to editors and reviewers. We strongly encourage code deposition in a community repository (e.g. GitHub). See the Nature Portfolio guidelines for submitting code & software for further information.

Data

Policy information about availability of data

All manuscripts must include a data availability statement. This statement should provide the following information, where applicable:

- Accession codes, unique identifiers, or web links for publicly available datasets
- A description of any restrictions on data availability
- For clinical datasets or third party data, please ensure that the statement adheres to our policy

The raw sequencing data generated in this study have been deposited in the NCBI database under accession code BioProject ID PRJNA1240917 [https://www.ncbi.nlm.nih.gov/bioproject/PRJNA1240917]. The crystal structure of chloroquine bound to sapB are available under PDB accession code 4V2O [https://doi.org/10.2210/pdb4V2O/pdb]. All other data supporting the findings of this study are available within the paper and its Supplementary Information. Source data are provided with this paper.

Research involving human participants, their data, or biological material

Policy information about studies with human participants or human data. See also policy information about sex, gender (identity/presentation), and sexual orientation and race, ethnicity and racism.

Reporting on sex and gender	n/a.
Reporting on race, ethnicity, or other socially relevant groupings	n/a.
Population characteristics	n/a.
Recruitment	n/a.
Ethics oversight	n/a.

Note that full information on the approval of the study protocol must also be provided in the manuscript.

Field-specific reporting

Please select the one below that is the best fit for your research. If you are not sure, read the appropriate sections before making your selection.

[x] Life sciences [ ] Behavioural & social sciences [ ] Ecological, evolutionary & environmental sciences

For a reference copy of the document with all sections, see nature.com/documents/nr-reporting-summary-flat.pdf

Life sciences study design

All studies must disclose on these points even when the disclosure is negative.

Sample size	Sample sizes had at least n = 3 biological replicates unless otherwise noted and no statistical methods were used to predetermine sample size. The details of the sample size are provided in the figure legends and where relevant.
Data exclusions	No data were excluded.
Replication	Experiments were repeated and experimental findings were reproducible.
Randomization	All samples or organisms were randomly allocated into experimental groups.
Blinding	Investigators were not blinded to group allocation during data collection and analysis as no subjective data collection or scoring was required.

Reporting for specific materials, systems and methods

We require information from authors about some types of materials, experimental systems and methods used in many studies. Here, indicate whether each material, system or method listed is relevant to your study. If you are not sure if a list item applies to your research, read the appropriate section before selecting a response.

Materials & experimental systems

n/a	Involved in the study
☒	☐ Antibodies
☐	☒ Eukaryotic cell lines
☒	☐ Palaeontology and archaeology
☐	☒ Animals and other organisms
☒	☐ Clinical data
☒	☐ Dual use research of concern
☒	☐ Plants

Methods

n/a	Involved in the study
☒	☐ ChIP-seq
☐	☒ Flow cytometry
☒	☐ MRI-based neuroimaging

Eukaryotic cell lines

Policy information about cell lines and Sex and Gender in Research

Cell line source(s)	Human embryonic kidney 293T cells (C6008), human cervical cancer HeLa cells (C6330), and the monocytic leukemia THP-1 cells (C6960) were purchased from Beyotime. 293T cells stably expressing the luciferase reporter (293T-Luc2-tdT, 1101HUM-PUMC000622) were obtained from the National Biomedical Cell-Line Resource.
Authentication	The morphology of cells were checked at every passage for authentication purposes.
Mycoplasma contamination	Cells were subjected to mycoplasma testing prior to use.
Commonly misidentified lines (See ICLAC register)	No commonly misidentified lines were used in this study.

Animals and other research organisms

Policy information about studies involving animals; ARRIVE guidelines recommended for reporting animal research, and Sex and Gender in Research

Laboratory animals	C57BL/6 mice aged between 6 and 8 weeks and Ai9 mice aged between 6 and 9 weeks were used in this study. Mice were housed under standard conditions (12 h light and 12 h dark cycles, 20–22 °C, 40–60% humidity) with food and water provided ad libitum.
Wild animals	No wild animals were used in this study.
Reporting on sex	Female for C57BL/6 mice and male for Ai9 mice. Sex was not considered in the study design.
Field-collected samples	No samples were collected from the field.
Ethics oversight	Animal care and experimental protocols were approved by the Institutional Animal Care and Use Committee of the First Affiliated Hospital of Southern University of Science and Technology (protocol number LL-KY-2020283) in accordance with the guidelines for the care and use of laboratory animals.

Note that full information on the approval of the study protocol must also be provided in the manuscript.

Plants

Seed stocks	n/a.
Novel plant genotypes	n/a.
Authentication	n/a.

Flow Cytometry

Plots

Confirm that:

- [x] The axis labels state the marker and fluorochrome used (e.g. CD4-FITC).
- [x] The axis scales are clearly visible. Include numbers along axes only for bottom left plot of group (a 'group' is an analysis of identical markers).
- [x] All plots are contour plots with outliers or pseudocolor plots.
- [x] A numerical value for number of cells or percentage (with statistics) is provided.

Methodology

Sample preparation	Samples were prepared as described in the "Methods" section.
Instrument	Agilent NovoCyte
Software	NovoExpress software
Cell population abundance	10,000 events were collected and analyzed.
Gating strategy	FSC-A/SSC-A and FSC-A/FSC-H were applied to excluded debris and cell-cell complexes, respectively. PBS-treated cells were used to differentiate positive and negative events.

- [x] Tick this box to confirm that a figure exemplifying the gating strategy is provided in the Supplementary Information.
